# Varietal Effect on Composition and Digestibility of Seedless Table Grapes (*Vitis vinifera* L.) under In Vitro Conditions

**DOI:** 10.3390/foods11243984

**Published:** 2022-12-09

**Authors:** Marianela Desireé Rodríguez, Joaquin García-Cordero, Diana Suárez-Coca, Maria Luisa Ruiz del Castillo, Gracia Patricia Blanch, Sonia de Pascual-Teresa

**Affiliations:** 1 Institute of Food Science, Technology and Nutrition (ICTAN), Spanish Research Council (CSIC), Jose Antonio Novais 6, 28040 Madrid, Spain; 2 Facultad de Ciencias Agropecuarias, Universidad Nacional de Córdoba, Córdoba 5001, Argentina

**Keywords:** grape, variety, phenolics, antioxidant, digestion

## Abstract

Grapes are one of the richest sources of polyphenols in the Mediterranean diet and, therefore, a very good source of antioxidants in the human diet. For practical reasons, in recent years the market for seedless grape varieties has grown exponentially. These varieties are not well characterized yet, and therefore it is necessary to study the changes in composition that these new varieties bring in. Likewise, the effect of digestion on the bioavailability of polyphenols in foods of plant origin is well known, which, consequently, will also affect antioxidant activity and, in general, bioactivity. In this work, we studied the effect of the grape variety on the initial grape composition and on the absorbable fraction, as it would reach the intestine after in vitro digestion. Our results showed that black and red varieties have great potential for increasing the antioxidant content of the diet. Additionally, we have concluded that all polyphenols, with the exception of flavanols, see their bioavailable fraction diminished under in vitro conditions.

## 1. Introduction

Grapes (*Vitis vinifera* L.) are a major fruit crop all around the world with many different cultivars varying in flavor and color. In addition to their sensory characteristics, grapes and their derived products, such as wine, are known for their high antioxidant power, which is associated with their high polyphenolic compound content. On this matter, grapes are one of the most important sources of antioxidants compared to other fruits and vegetables [1]. Polyphenols are known to play an important role in human health, mainly as antioxidants, either by exerting a protective effect directly in the human body or by protecting other nutrients from being oxidized and degraded.

On the other hand, the basic characteristic of current table grape production is its adaptation to consumer requirements to improve grape quality. In recent years, an attribute that has come to be considered important in grape quality is the absence of seeds. Seedless grapes are bred by propagation, through the implantation of parts of the vine with the genetic defect that causes seedless grapes to grow. There are compositional studies on grape berries in the literature. However, most of them have been carried out on wine grape varieties or table varieties that retain their seeds [2,3].

Additionally, it has been shown that the effect of digestion on the bioavailability and thus bioactivity of food polyphenols is crucial [4]. So far, the INFOGEST method for digestibility assessment has proven to be the most widely accepted method available, despite some limitations that make difficult the extrapolation of results for the purposes of improving human or animal bioavailability [5]. This is why we have decided to use it for the purpose of determining the digestibility of the polyphenols of seedless grapes in this work.

Because the composition of polyphenols and other bioactive compounds in plant foods varies widely, both in quantity and in detailed composition, it is essential to carry out a detailed identification and quantification of polyphenols in each individual plant food, taking into account its variety and other agronomic parameters and post-harvest treatments that could influence their composition [6].

On the other hand, the irruption of the health-related potential of many foods with polyphenolic content has pointed out the importance of knowing not only the detailed composition of a specific plant food product, but also studying the effect that digestion could have on them. This is because not all polyphenols have the same effect in humans and because digestion can modify not only the quantitative polyphenolic profile of plant food but also its qualitative profile. Consequently, the digestion process would greatly influence the final effect of that food product [4].

Therefore, the purpose of this research was to establish compositional differences in polyphenolic profiles and the antioxidant power of different varieties of seedless table grapes. Additionally, we will study how these differences in variety and composition might affect the final intestinal bioaccessible polyphenolic fraction.

## 2. Materials and Methods

### 2.1. Materials

Seedless table grapes of six varieties were used: Autumn Crisp and Pristine (white varieties), Scarlotta and Crimson (red varieties) and Adora and Melody (black varieties). All of them were cultivated in Murcia (Spain) and kindly supplied by MOYCA, (Totana, Murcia). Grape berries were collected at the commercial maturity stage and sent to ICTAN on the same day under refrigeration. On the day of arrival, grapes were manually separated from the stems and kept at −80 °C until analysis. For polyphenol extraction and analysis, grapes were used after having been freeze-dried (VirTis Benchtop-6KB). For digestions and subsequent analysis and determinations, grapes were directly used after defrosting.

### 2.2. Polyphenol Extraction

For the detailed polyphenolic characterization of the grapes, an amount of 2 g of lyophilized grapes was extracted with 25 mL of MeOH/H_2_O (70:30 *v*/*v*). The samples were then homogenized in Ultra-Turrax (T18 Digital, IKA) at up to 20.000 rpm for 5 min. The extraction was performed using a sonication water bath for 10 min. The supernatant was collected and the residue was re-extracted following the same procedure. The collected supernatants were pooled and concentrated using a vacuum rotary evaporator (R-210, BUCHI SWITZERLAND). All extractions were performed in triplicate.

The sample extraction for digestion samples was performed as previously described [7]. Briefly, 0.3 mL digestion media were placed in an Eppendorf and suspended in 0.7 mL MeOH (acidified with formic acid 0.1%). The digestion samples were then vortexed and sonicated for 15 min. The samples were centrifuged at 10,000 rpm for 15 min at 4 °C and the supernatant was collected. The residue was re-suspended in 0.5 mL of MeOH/H_2_O (70:30 *v*/*v* acidified with formic acid 0.1%) and re-extracted following the same procedure twice. Supernatants were combined and filtered (0.45 μm).

### 2.3. Static In Vitro Digestion

The simulated in vitro digestion model was prepared using the protocol described by INFOGEST, which includes oral, gastric and intestinal phases [5]. The grape varieties were digested, and a control tube was made to have a digestion blank (without the sample, only digestion reagents). Briefly, 2 g of whole grapes were transferred to a volumetric flask at a 1:1 ratio with simulated salivary fluid, and salivary amylase (75 U/mL of final mixture, A1031 Sigma). This mixture was digested for 2 min at 37 °C using a shaking dry bath. The pH was immediately adjusted to 3 with 5 M HCl, to stop the action of amylase. The oral bolus was then diluted 1:1 with simulated gastric fluid in the presence of pepsin (2000 U/mL of a final mixture, P7012 Sigma). The mixture obtained was subsequently incubated in a shaking dry bath for 2 h at 37 °C. The reaction was stopped by raising the pH to 7 with simulated intestinal fluid and 5 M NaOH. The intestinal phase was performed by adding pancreatin from porcine pancreas (100 U trypsin activity/mL of a final mixture, P7545 Sigma) and porcine bile extract (10 mM, B8631 Sigma) in simulated intestinal fluid. The intestinal phase was carried out by mixing the gastric digest with the same volume of intestinal fluids and enzymes. Finally, the intestinal phase was stopped by heating for 15 min at 85 °C. Aliquots of 0.5 mL were removed in the oral and gastric phases. Intestinal digest was centrifuged at 5000 rpm for 10 min, and the supernatants were placed in 2 mL Eppendorf. Aliquots (oral, gastric and intestinal phase) were stored at −20 °C until analysis. Digests were performed in duplicate.

### 2.4. Total Polyphenol Content (TPC) and Antioxidant Activity (DPPH and FRAP Methods)

TPC was determined by the Folin–Ciocalteu method in 96-well plates by using a BioTek Synergy HT multi-mode microplate reader with BioTek’s Gen5TM software (BioTek Instruments Inc., Winooski, VT, USA). Data were expressed as gallic acid equivalents. The antioxidant and antiradical power of the grape samples as well as the digestion products in the different phases were determined by two methods, the DPPH method and the FRAP method. Both methods were executed in the same way as the previous method, in 96-well plates and using the same microplate reader as before [8]. All analyses were performed in triplicate and in every case, average and standard deviation values were calculated.

### 2.5. HPLC-QTOF-MS Analysis of the Seedless Table Grapes

The identification of the different compounds in the six varieties of seedless table grapes was carried out by HPLC-MS- QTOF using an HPLC (Agilent 1200, Agilent Technologies) with a quaternary pump (G1311A) coupled with a diode array detector (Agilent G1315B) and an Agilent 6530 Accurate-Mass QTOF LC/MS with Electrospray Ionization (ESI) (Agilent Technologies). The column was a Phenomenex Luna C18 column (3 μm, 4.6 mm × 150 mm), set thermostatically to 25 °C.

A gradient between solvent A (0.1% formic acid in water) and solvent B (0.1% formic acid in acetonitrile) was applied (10% B at 0 min, 30% B at 30 min, 35% B at 35 min, 40% B at 45 min, 10% B at 50 min and 10% at 60 min) at a flow rate of 0.5 mL/min. Injection volume was 5 μL. MS conditions were as in [8]. For the identification and quantification of compounds, MS and tandem mass spectrometry fragmentation spectra (MSMS) experiments were carried out with a collision energy of 20 V. Data acquisition and processing were performed with the Masshunter Data Acquisition B.05.01 and Masshunter Qualitative Analysis B.07.00 SP2 software. Compounds were identified by comparing mass spectra and retention time with the corresponding standard if available.

### 2.6. Quantitative HPLC-DAD Analysis of the Digestion Products

The quantification of the different compounds in the six varieties of seedless table grapes after oral, gastric and intestinal digestion was executed using HPLC-DAD on an Agilent 1200 Series liquid chromatography system equipped with a quaternary pump and a photodiode array detector (DAD) (Agilent Technologies, Waldrom, Germany). The column was a Phenomenex Luna C18 column (3 μm, 4.6 mm × 150 mm), set thermostatically to 25 °C.

Chromatographic data were acquired and processed using an Agilent Chemstationfor LC 3D system (Rev. B.04.01) (Agilent Technologies). Briefly, the binary mobile phase used for analyses was aqueous 0.1% formic acid in water (A) and 0.1% formic acid in HPLC-grade acetonitrile (B) at a flow rate of 0.5 mL/min. The elution was started with 10% B, and the gradient was 32% B from 0 to 30 min, 36% B from 30 to 35 min and 47% B from 35 to 40 min, followed by an additional 5 min isocratically at 47% B and 10 min column stabilization at 10% B prior to the next analysis. Blanks were injected every 5 analyses to ensure good column cleaning and a mixture of standards was injected every 10 analyses to ensure good quantification consistency.

Detection wavelengths were 280, 320 and 520, which were, respectively, used for flavanol, flavonol and anthocyanin quantification as detailed below. Compounds were quantified by comparing UV-Vis spectra and retention time with the corresponding standard if available and taking into consideration the identifications performed using the methodology described previously.

### 2.7. Statistical Analysis

Data analysis was carried out with IBM SPSS version 27 statistical software (SPSS, Inc., Chicago, IL, USA, 2020). The level of significance was set at *p* < 0.05. Values are expressed as mean and standard deviation (M ± SD). Data were examined for normal distribution with the Kolmogorov–Smirnov test. As a result of the normality test, a repeated measure ANOVA was applied when comparing the means of the results of TPC, DPPH, FRAP and the quantitative composition by HPLC-DAD in the six different varieties of seedless grape extracts. Moreover, another ANOVA was performed to compare the different results of TPC, DPPH, FRAP and the quantitative composition by HPLC-DAD at each stage of the in vitro digestion in each variety of seedless grape.

## 3. Results

### 3.1. Seedless Table Grapes (Vitis vinifera *L.*) Characterization

After seedless grapes of the varieties Autumn Crisp and Pristine (white varieties), Scarlotta and Crimson (red varieties) and Adora and Melody (black varieties) were extracted and analyzed by HPLC-QTOF-MS, both in the positive and negative modes, the qualitative composition of the different varieties was determined (Table 1).

The black varieties, Adora and Melody, were remarkably rich in anthocyanins, showing Delphinidin, cyanidin, petunidin and malvidin glucosides as well as derivatives with coumaric acid.

Flavonols such as quercetin and kaempferol and their glycosides, and flavanols, such as epicatechin or epigallocatechin, were present in all seedless table grapes analyzed. Furthermore, we detected a peak at 8.2 min showing an absorbance of 280 nm, a molecular ion in the positive mode at 205.0954 and a fragment in the MSMS at 146.0598. After comparison with an authentic standard, this was identified as the amino acid tryptophan.

Seedless grape extracts were also analyzed by HPLC-DAD in order to quantitatively characterize their composition. Anthocyanins were quantified at a wavelength of 520 nm, flavonols at 360 nm and compounds such as tryptophan, gallic acid and flavanols at a UV wavelength of 280 nm. The standards used for the subsequent quantification were cyanidin-3-glucoside (for the quantification of anthocyanins), quercetin-3-glucoside (for flavonols), catechin (flavanols), the amino acid tryptophan and the phenolic acid gallic acid.

The results shown in Table 2 indicate that malvidin-3-glucoside is the most abundant anthocyanin in Melody seedless grapes, while malvidin-3-coumaroylglucoside is the most abundant anthocyanin in Adora grapes. In all, black seedless grapes are characterized by a high proportion of malvidin derivatives, and red seedless grapes by the relatively high contents of peonidin-3-glucoside. The red seedless grape Scarlotta, on the other hand, showed higher levels of the flavonols quercetin-3-glucuronide and quercetin-3-glucoside.

As mentioned before, tryptophan was detected and quantified in all six seedless grape varieties, showing the highest levels in Crimson red grapes and the lowest in Pristine white grapes.

Additionally, TPC by the Folin–Ciocalteu method and antioxidant activity by the two selected methods, DPPH and FRAP, were determined in the same extracts and the results are shown in Table 3. Red (Scarlotta and Crimson) and black (Adora and Melody) varieties showed higher antioxidant power both in the DPPH and the FRAP method.

Total polyphenol contents were also higher in the same grape varieties, the highest content being that of Scarlotta variety followed by the Melody variety, with no significant differences between these two seedless grapes.

### 3.2. Effect of In Vitro Digestion on Seedless Table Grape (Vitis vinifera *L.*) Composition

Fresh grapes of the varieties Autumn Crisp and Pristine (white varieties), Scarlotta and Crimson (red varieties) and Adora and Melody (black varieties) were subjected to an in vitro digestion process under the conditions described in the methodology section. Samples were collected throughout the digestion process. The different aliquots collected from the digestions were extracted and analyzed by HPLC-DAD in order to quantify the different compounds present in the seedless grapes and their evolution throughout the digestion process.

**Figure 1:**
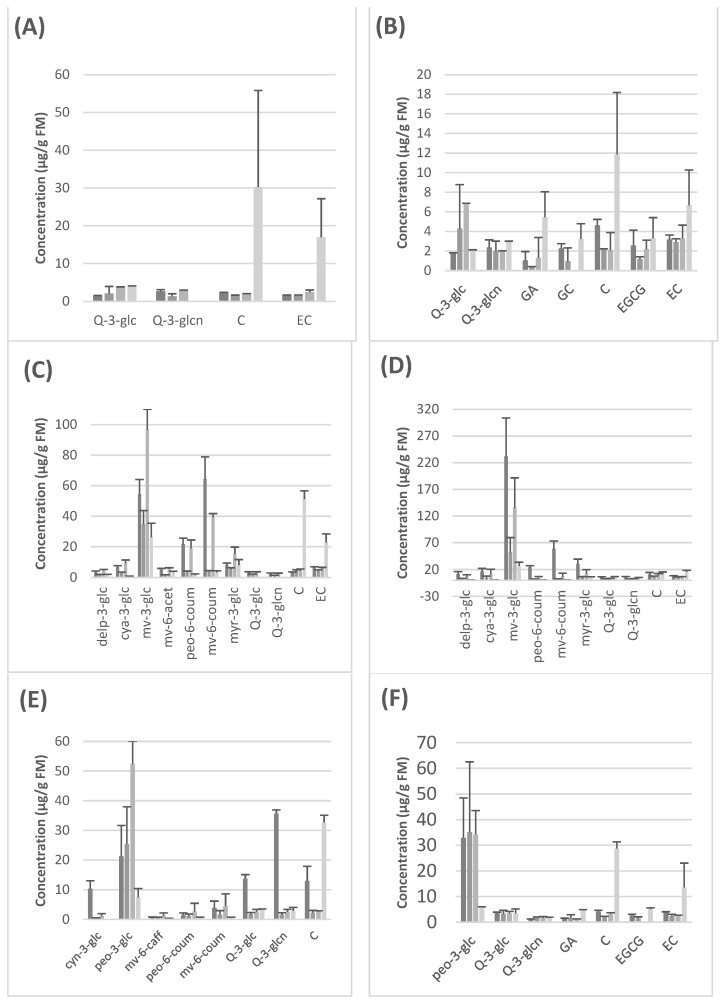
Concentrations of the most representative compound in each seedless grape at each stage of digestion. (**A**) Autumn Crisp, (**B**) Pristine, (**C**) Adora, (**D**) Melody, (**E**) Scarlotta, (**F**) Crimson. ■ Raw material, ■ Oral phase, ■ Gastric phase, ■ Intestinal phase. FW: fresh weight, Delp-3-glc: delphinidin-3-glucoside, cya-3-glc: cyanidin-3-glucoside, mv-3-glc: malvidin-3-glucoside, mv-6-acet: malvidin-3-(6-acetylglucoside), mv-6-caff: malvidin-3-(6-caffeoylglucoside), peo-6-coum: peonidin-3-(6-coumaroylglucoside), mv-6-coum: malvidin-3-(6-coumaroylglucoside), myr-3-glc: myricitin-3-glucoside, Q-3-glc: quercetin-3-glucoside, Q-3-glcn: quercetin-3-glucoronide, GA: gallic acid, GC: gallocatechin, C: catechin, EGCG: epigallocatechin, EC: epicatechin. Data expressed as mean + standard error bar.

Anthocyanins were quantified at a wavelength of 520 nm, flavonols at 360 nm and compounds such as tryptophan, gallic acid and flavanols at a UV wavelength of 280 nm. The standards used for the subsequent quantification were cyanidin-3-glucoside (for the quantification of anthocyanins), quercetin-3-glucoside (for flavonols), catechin (for flavanols), the amino acid tryptophan and the phenolic acid gallic acid. Figure 1 and Supplementary Materials Tables S1–S6, show the quantitative results obtained for each seedless grape variety after analysis by HPLC-DAD.

Additionally, the antioxidant activity was measured in every digestion phase in order to establish the possible effect of digestion, in our conditions, on the antioxidant power of the different seedless grape varieties. In every case TPC, FRAP and DPPH were measured in the same extracts as for the HPLC-DAD-MS analysis. One should note that the use of different methods in parallel for the calculation of the antioxidant power of a given food is advisable. In this case, we used FRAP to determine the ferric-reducing power of the different digestion phases, DPPH in order to establish the antiradical capacity of the digestion products and TPC as a general method widely accepted for the determination of polyphenols in food samples.

Results are shown in Table 4 and Table 5 and reflect a significant decline in the antioxidant potential of seedless grapes with digestion, already starting as of the oral phase. Interestingly the results show a slight increase in antioxidant power after the intestinal phase in relation to the oral and gastric phases.

## 4. Discussion

The results encountered when analyzing the different extracts of seedless grapes are in line with previous work on both table and wine grapes, with or without seeds [9]. However, we did not find any diglucoside of anthocyanin or any pelargonidin in the red or black seedless grapes. Interestingly, we did find a significant difference in the anthocyanin composition between the red and black varieties. Malvidin-3-glucoside was the main anthocyanin in Adora, and more markedly in Melody, black seedless grapes. While Peonidin-3-glucoside was the main anthocyanin in Scarlotta and Crimson red seedless grapes. Additionally, a high content of malvidin-3-coumaroylglucoside was found for both black seedless grape varieties, Adora and Melody.

The levels of the flavanols, catechin and epicatechin were also higher in the black seedless grape variety Melody in comparison with the rest of the seedless grapes. Within flavonols, the main compound found was for all samples quercetin-3-glucoside, the red seedless grape variety Scarlotta being the one with the highest content.

Grape polyphenols have been studied deeply in the past by different authors and our results are in line with other reports [10]. An interesting finding is that malvidin-3-glucoside was the main anthocyanin in the black seedless varieties, while peonidin-3-glucoside is the main anthocyanin in the red seedless varieties. In all, the highest total concentration of polyphenols calculated as the addition of all the integrated individual peaks is as high as 417 µg/g FW in the black seedless grape Melody. These values are considered high for a table grape and are in line with other anthocyanin-rich foods such as blueberry or elderberry [11].

In another work by Crupi et al. with the aim of characterizing the polyphenolic composition of seedless table grapes skin extracts from different varieties grown in southern Italy, the authors showed that, in their case, the main anthocyanins were also malvidin-3-glucoside and petunidin-3-glucoide, depending on the variety [12]. The only common variety from the work of Crupi et al.’s and ours was Crimson, and the results obtained are similar. However, one interesting finding from that work was that they found important quantitative differences between years in the amounts of individual anthocyanins accounting for as much as five to six times from one year to another. In this same line, other authors have shown the effect of maturation on the aroma and polyphenolic individual and total content of seedless grapes [13]. According to this work, total phenolics increased to a maximum in the ripe grapes, while flavanols and stilbenes decreased.

On the other hand, we have reported high levels of tryptophan that are in the same range as the ones shown by other authors in Verdejo grapes under different agronomical conditions [14]. With this in mind, it might be that the difference found between, for instance, Crimson grapes (338.76 ± 28.03) and Pristine grapes (16.86 ± 1.31), is due to differences in agronomical conditions or the state of maturation at which the grapes were collected. It is well known that tryptophan concentrations in grapes increase with maturation and that the tryptophan metabolic pathway is implicated at different levels in the development of grape aroma [15]. However, in our study, all seedless grapes were of a very similar origin, from the same geographical area, harvested at the same time of the year and at the same maturity stage. In accordance with this, and as far as we know, we could conclude that the differences encountered in tryptophan might be due to the seedless grape variety itself.

In general, Scarlotta grapes were the ones showing the highest TPC, as measured by the Folin–Ciocalteu method, but with no significant differences from Melody grapes. In parallel, Scarlotta grapes were also the ones showing the highest FRAP and DPPH values, referred to as gallic acid equivalents. This could be due to the fact that the Scarlotta variety is the one that presents the highest concentrations of quercetin derivatives, since this polyphenol stands out for its high antioxidant activity in both the DPPH and FRAP assays, superior to other polyphenols such as gallic acid or resveratrol [16]. However, it might also be explained by possible interactions between different polyphenols or non-polyphenols present in grapes [8]. As a whole, the black seedless varieties Adora and Melody are the richest in anthocyanins and the ones showing a higher antioxidant effect, given that they show the highest TPC and antioxidant activity at the end of the digestion process. However, there is a slight inconsistency between the polyphenolic content calculated by the individual quantification of, mainly, flavonoids and the TPC values. This might be because the HPLC-Qtof analysis for identification and thus the HPLC-DAD analysis for quantification were performed in the positive ionization mode and it is known that phenolic acids and other polyphenols are better ionized in the negative mode. Moreover, these changes are in agreement with the results encountered in another seedless grape variety, Superior Seedless, showing a contradictory maturation effect between TPC and individual flavanols and stilbenes [13].

Food digestibility and the use of in vitro models to study it have garnered much attention from the scientific community in the last years. Food bioactives, like polyphenols, are important food components that might have an effect on human health that goes beyond the nutritional value of that food. However, for food bioactives to achieve their beneficial effect, they need to be able to access the target tissue. In this sense, the so-called digestibility, should be the first critical step once the food is ingested. Digestion might decrease and/or transform the bioactive compound, even making it more or less susceptible to absorption once it arrives in the intestine. Nowadays, the INFOGEST in vitro model for digestion, with all its limitations, is widely used in order to study the digestibility of food components or food bioactives [5]. We report here for the first time the effect of digestion on grape polyphenols in this in vitro model. In all, the oral phase produces a decrease in the concentration of all polyphenols. However, the gastric phase causes an increase in some anthocyanins, more precisely in malvidin-3-glucoside (Figure 1C,D) and peonidin-3-glucoside (Figure 1E,F). This effect might be explained by the drop in pH due to the gastric ingestion conditions in this model, since anthocyanins exist basically in their cationic form only at a pH level below 2, and as pH increases, there is a rapid deprotonation, producing quinonoidal forms that cannot be detected under our conditions [17].

On the other hand, we observed an increase in catechin and epicatechin levels during intestinal digestion that was particularly evident in the case of the two white seedless grapes, Autumn Crisp and Pristine (Figure 1A,B). This effect is due to the breakdown of more polymerized flavan-3-ols such as B-type procyanidin dimers, trimers, tetramers, etc. which have been detected in grapes by other authors and that, under the INFOGEST conditions, might produce catechin and epicatechin as monomeric units [5,18]. In all, the qualitative polyphenolic composition changes found in our work might explain the differences in the TPC because, as has been largely shown, different individual polyphenols, as well as other interferents, might react differently with the Folin–Ciocalteu reactive [19,20].

## 5. Conclusions

All things considered, we can conclude that seedless grapes in general could be a good source of antioxidant polyphenols in the human diet. The black seedless grape varieties, Adora and Melody, are a very good source of anthocyanins and, in general, of polyphenols and antioxidants. In this sense, the main limitation of our work is that we worked only with just one-year grape harvesting, and thus we omitted the potential effect of maturation and environmental and agronomical conditions on the varietal changes in antioxidant and polyphenolic composition.

On the other hand, the digestion of the different seedless grape varieties decreased the amounts of polyphenols susceptible to absorption in the intestine in the present in vitro conditions, mostly anthocyanins and flavonols. However, the case of catechin and epicatechin might be an exception and therefore the absorption of flavan-3-ols (proanthocyanidins) might be increased by the digestion process. Undoubtedly, this result needs to be further investigated, in order to better prove if the increase in the accessibility of the absorption of flavanols could be extrapolated to in vivo conditions as well as to establish if this effect is maintained in other food matrices.

## Figures and Tables

**Table 1:** Seedless grapes composition by HPLC-QTOF-MS.

Compound	Rt	M+	M-	*Autumn Crisp*	*Pristine*	*Adora*	*Melody*	*Scarlotta*	*Crimson*
gallic acid	5.3		169.0149	+	+		+	+	+
Gallocatechin	7.3		305.0672		+	+	+		
delphinidin-3-glucoside	7.7	465.1005				++	++		+
Tryptophan	8.2	205.0954		++	+	+	+	++	+
cyanidin-3-glucoside	9.8	449.1099				+	+	+	+
procyanidin B1	9.9		577.1367	+	+	+	+	+	+
petunidin-3-glucoside	10.6	479.1169				+	++	+	+
pelargonidin-3-glucoside	11.7	433.11.1		+	+	+	+	+	+
Catechin	12.4		289.0733	+	+	+	+	+	+
peonidin-3-glucoside	12.6	463.1217				++	++	++	+
malvidin-3-glucoside	13.0	493.1326				++	++	++	+
Epigallocatechin	13.8		305.0808		+		+	+	
Epicatechin	15.8		289.0731	+	+	+	+	+	
quercetin 3,7-diglucoside	18.2	627.1552		+	+			+	+
myricetin-3-glucoside	19.8		479.0848			+	+		+
malvidin-3-acetylglucoside	20.4	535.1433				++	+		+
delphinidin3(6coumaroylglucoside)	22.0	611.1399				+	+		
rutin (quercetin-3-rutinoside)	22.4	611.1611			+				+
malvidin 3-caffeoylglucoside	23.5	655.1655				+	+	+	+
quercetin-3-glucoside	23.8		463.0892	+	+	+	+	+	+
cyanidin 3-(6-coumaroylglucoside)	24.0	595.1440				+	+		+
quercetin-3-glucuronide	24.5		477.0684	+	+	+	+	+	+
peonidin 3-(6-coumaroylglucoside)	25.3	609.1597				+	+	+	+
malvidin 3-(6-coumaroylglucoside)	26.8	639.1705				++	++	+	+
kaempferol-3-glucoside	27.2		447.0942	+	+	+	+		+
quercetin	38.8		301.0340	+	+		+		+
kaempferol	44.1	611.1399	285.0442	+			+		+

+ area inferior to 10 million, ++ area superior to 10 million.

**Table 2:** Seedless grapes quantitative composition (mean ± SD, µg/g FW) *.

Compound	*Rt*	*Autumn Crisp*	*Pristine*	*Adora*	*Melody*	*Scarlotta*	*Crimson*	*Sig*
gallic acid	5.7	0.38 ± 0.06	1.06 ± 0.89		0.72 ± 0.1	0.76 ± 0.24	1.17 ± 0.38	0.286
gallocatechin	8.7		2.28 ± 0.48					
delphinidin-3-glucoside	9.4			3.35 ± 0.97 ^b^	11.37 ± 4.73 ^a^	0.68 ± 0.17 ^b^		0.008
triptophan	9.5	94.50 ± 15.26 ^b^	16.86 ± 1.31 ^c^	64.91 ± 2 ^b,c^	91.02 ± 36.15 ^b^	47.86 ± 9.4 ^b,c^	338.76 ± 28.03 ^a^	<0.001
cyanidin-3-glucoside	12.0			6.37 ± 1.21 ^a^	15.85 ± 6.14 ^b^	1.46 ± 2.67 ^a^	3.07 ± 0.56 ^a^	0.009
procyanidin B1	12.5	1.37 ± 0.3 ^a^	3.66 ± 1.01 ^a,b^		5.01 ± 1.64 ^b^			0.02
catechin	13.5	2.24 ± 0.18 ^c^	4.63 ± 0.6 ^b,c^	3.60 ± 0.06 ^b,c^	10.75 ± 4.01 ^a,b^	12.93 ± 4.92 ^a^	4.17 ± 0.46 ^b,c^	0.001
peonidin-3-glucoside	14.0			4.71 ± 0.85 ^b^	12.18 ± 3.79 ^a,b^	21.25 ± 10.39 ^a,b^	32.88 ± 15.59 ^a^	0.014
malvidin-3-glucoside	14.7			54.20 ± 9.86 ^b^	231.44 ± 72.20 ^a^	12.47 ± 6.09 ^c^	11.86 ± 5.62 ^c^	<0.001
epigallocatechin	15.4		2.56 ± 1.58				2.7 ± 0.38	0.884
epicatechin	16.7	1.6 ± 0.11 ^c^	3.19 ± 0.45 ^b,c^	6.7 ± 0.14 ^a^	7.16 ± 1.55 ^a^		3.93 ± 0.12 ^b^	<0.001
myricetin-3-glucoside	20.5			7.89 ± 1.47 ^b^	30.23 ± 9.12 ^a^			0.014
malvidin-3-acetylglucoside	21.9			4.75 ± 1.1 ^b^	8.92 ± 1.63 ^a^			0.021
delphinidin-3-(6-coumaroylglucoside)	23.5			1.97 ± 0.47				
malvidin-3-caffeoylglucoside	24.3			2.31 ± 0.7 ^a^	1.41 ± 0.27 ^a,b^	0.49 ± 0.28 ^b^		0.015
quercetin-3-glucoside	25.3	1.39 ± 0.17 ^b^	1.74 ± 0.08 ^b^	2.89 ± 0.87 ^b^	4.75 ± 1.95 ^b^	13.68 ± 1.39 ^a^	3.4 ± 0.49 ^b^	<0.001
cyanidin-3-(6-coumaroylglucoside)	25.8			3.34 ± 0.95				
quercetin-3-glucoronide	26.0	2.66 ± 0.46 ^b^	2.36 ± 0.79 ^b^	2.03 ± 0.89 ^b^	4.69 ± 2.54 ^b^	35.68 ± 1.22 ^a^	1.09 ± 0.14 ^b^	<0.001
peonidin-3-(6-coumaroylglucoside)	26.6			21.62 ± 4.04 ^a^	23.81 ± 3.11 ^a^	1.46 ± 0.68 ^b^		<0.001
malvidin-3-(6-coumaroylglucoside)	27.5			64.25 ± 14.56 ^a^	58.36 ± 14.99 ^a^	3.92 ± 2.23 ^b^		<0.001
kaempferol-3-glucoside	29.0				1.56 ± 0.29			

* All analyses were performed in triplicate. Results are shown as mean ± standard deviation. ^a^, ^b^, ^c^: different letters indicate significant differences between grapes varieties.

**Table 3:** Seedless grape antioxidant activity.

	*Autumn Crisp*	*Pristine*	*Adora*	*Melody*	*Scarlotta*	*Crimson*	*Sig*
DPPH (µmol Trolox/g FW)	6.2 ± 0,4 ^d^	7.77 ± 0.56 ^c,d^	17.78 ± 1.88 ^b^	20.34 ± 1.94 ^b^	32.3 ± 0.2 ^a^	11.3 ± 0.96 ^c^	<0.001
FRAP (µmol Trolox/g FW)	6.67 ± 0.37 ^d^	7.25 ± 0.85 ^d^	19.16 ± 0.97 ^b,c^	25.73 ± 6.1 ^a,b^	35.4 ± 5.89 ^a^	12.2 ± 2.06 ^c,d^	<0.001
TPC (mg GAE/g FW)	1.1 ± 0.14 ^c^	1.25 ± 0.22 ^c^	3.1 ± 0.14 ^b^	4.39 ± 0.01 ^a^	5.5 ± 0.57 ^a^	2.85 ± 0.21 ^b^	<0.001

Data expressed as mean ± standard deviation from triplicates. ^a^, ^b^, ^c^, ^d^: different letters indicate significant differences between grape varieties. FW: fresh weight, GAE: Gallic acid equivalent.

**Table 4:** Seedless grape antioxidant activity at each stage of digestion.

	DPPH (µmol Trolox/g FW)					FRAP (µmol Trolox/g FW)				
	Extraction	Oral	Gastric	Intestinal	Sig	Extraction	Oral	Gastric	Intestinal	Sig
***Autumn Crisp***	6.2 ± 0.4 ^a^	0.47 ± 0.09 ^b^	0.19 ± 0.03 ^b^	1.23 ± 0.23 ^b^	<0.001	6.67 ± 0.37 ^a^	0.87 ± 0.12 ^b^	0.71 ± 0.15 ^b^	1.82 ± 0.26 ^b^	<0.001
***Pristine***	7.77 ± 0.56 ^a^	0.015 ± 0.006 ^b^	*	0.33 ± 0.19 ^b^	<0.001	7.25 ± 0.85 ^a^	0.44 ± 0.03 ^b^	0.82 ± 0.13 ^b^	1.76 ± 0.17 ^b^	<0.001
***Adora***	17.78 ± 1.88 ^a^	0.8 ± 0.02 ^b^	1.57 ± 0.36 ^b^	1.79 ± 0.37 ^b^	<0.001	19.16 ± 0.97 ^a^	1.34 ± 0.15 ^b^	3.19 ± 0.13 ^b^	3.62 ± 0.18 ^b^	<0.001
***Melody***	20.34 ± 1.94 ^a^	1.96 ± 0.06 ^b^	3.5 ± 0.21 ^b^	2.55 ± 0.27 ^b^	<0.001	25.73 ± 6.1 ^a^	2.45 ± 0.38 ^b^	4.5 ± 1.04 ^b^	4.16 ± 0.9 ^b^	0.003
***Scarlotta***	32.3 ± 0.2 ^a^	1.26 ± 0.14 ^b^	0.5 ± 0.13 ^b^	1.46 ± 0.28 ^b^	<0.001	35.4 ± 5.89 ^a^	1.9 ± 0.1 ^b^	2.52 ± 0.13 ^b^	2.77 ± 0.38 ^b^	<0.001
***Crimson***	11.3 ± 0.96 ^a^	1.06 ± 0.05 ^b^	1.4 ± 0.00 ^b^	1.26 ± 0.28 ^b^	<0.001	12.2 ± 2.06 ^a^	1.44 ± 0.03 ^b^	1.92 ± 0.3 ^b^	1.79 ± 0.38 ^b^	0.001

Data expressed as mean ± standard deviation. * Below detection point; ^a^, ^b^, ^c^: different letters indicate significant differences between grapes varieties. FW: fresh weight.

**Table 5:** Seedless grape polyphenol content at each stage of digestion.

	TPC (mg GAE/g FW)				
	Extraction	Oral	Gastric	Intestinal	Sig
***Autumn Crisp***	1.1 ± 0.14 ^a^	0.06 ± 0.01 ^b^	0.05 ± 0.01 ^b^	0.41 ± 0.29 ^a^	0.037
***Pristine***	1.25 ± 0.22	*	*	0.58 ± 0.31	0.13
***Adora***	3.1 ± 0.14 ^a^	0.18 ± 0.02 ^c^	0.55 ± 0.01 ^c^	1.2 ± 0.17 ^b^	<0.001
***Melody***	4.39 ± 0.01 ^a^	0.42 ± 0.04 ^c^	0.76 ± 0.08 ^c^	1.31 ± 0.28 ^b^	<0.001
***Scarlotta***	5.5 ± 0.57 ^a^	0.32 ± 0.02 ^b^	0.36 ± 0.04 ^b^	0.73 ± 0.24 ^b^	<0.001
***Crimson***	2.85 ± 0.21 ^a^	0.29 ± 0.04 ^b^	0.42 ± 0.03 ^b^	1.14 ± 0.57 ^b^	0.009

Data expressed as mean ± standard deviation. * Data below detection limit; ^a^, ^b^, ^c^: different letters indicate significant differences between grapes varieties. FW: fresh weight, GAE: Gallic acid equivalent.

## Data Availability

The data presented in this study are available in the article.

## Supplementary Materials

The following supporting information can be downloaded at: https://www.mdpi.com/article/10.3390/foods11243984/s1. Table S1. Concentrations of all compounds found in seedless grape Adora at each stage of digestion; Table S2: Concentrations of all compounds found in seedless grape Autumn Crisp at each stage of digestion; Table S3. Concentrations of all compounds found in seedless grape Pristine at each stage of digestion; Table S4. Concentrations of all compounds found in seedless grape Scarlotta at each stage of digestion; Table S5. Concentrations of all compounds found in seedless grape Crimson at each stage of digestion; Table S6. Concentrations of all compounds found in seedless grape Melody at each stage of digestion.
