# Supplementary material for: Varietal Effect on Composition and Digestibility of Seedless Table Grapes (Vitis vinifera L.) under In Vitro Conditions
Source: Foods. 2022 Dec 9;11(24):3984. doi: 10.3390/foods11243984 (PMC9777568; doi:10.3390/foods11243984)
Supplement: Supplementary file 1 [file foods-11-03984-s001.zip › foods-2000075-supplementary.pdf]

Table S1. Concentrations of all compounds found in seedless grape Adora at each stage of digestion.

Rt	*Compound*	*Raw material (µg/g FW)*	*Digestion (µg/g FW)*		
			Oral phase	*Gastric phase*	*Intestinal phase*
9.4	Delphinidin-3-glucoside	3.35 ± 0.97	1.80 ± 0.23	4.47 ± 0.82	2.12
9.5	Triptophan	64.91 ± 2	74.71 ± 0.69	125.43 ± 11.07	19900.30 ± 3551.79
12.0	Cyanidin-3-glucoside	6.37 ± 1.21	2.8 ± 0.72	9.42 ± 1.9	0.96
13.6	Catechin	3.60 ± 0.06	4.47 ± 0.66	4.57 ± 1.01	51.25 ± 5.39
14.0	Peonidin-3-glucoside	4.71 ± 0.85	*	*	*
14.7	Malvidin-3-glucoside	54.2 ± 9.86	34.78 ± 8.86	96.67 ± 13.35	26.26 ± 9.13
16.7	Epicatechin	6.70 ± 0.14	3.96 ± 1.07	5.88 ± 0.74	22.93 ± 5.46
20.6	Myricitin-3-glucoside	7.89 ± 1.47	5.4 ± 0.93	15.47 ± 4.27	8.04 ± 3.58
21.9	Malvidin-3-(6-acetylglucoside)	4.75 ± 1.1	1.5 ± 0.28	5.75 ± 0.59	3.48 ± 0.34
23.5	Delphindin-3-(6-coumaroylglucoside)	1.97 ± 0.47	1.2 ± 0.15	2.08 ± 1.05	*
24.3	Malvidin-3-(6-caffeoylglucoside)	2.31 ± 0.7	1.07 ± 0.28	5.52 ± 4.04	1.30 ± 0.34
25.3	Quercetin-3-glucoside	2.89 ± 0.87	1.87 ± 0.41	3.30 ± 0.47	*
25.8	Cyanidin-3-(6-coumaroylglucoside)	3.34 ± 0.95	0.12 ± 0.17	1.87 ± 0.48	1.24
26.0	Quercetin-3-glucoronide	2.03 ± 0.89	0.63 ± 0.89	2.49 ± 0.39	*
26.6	Peonidin-3-(6-coumaroylglucoside)	21.62 ± 4.04	3.22 ± 0.99	18.82 ± 5.6	1.56 ± 0.8
28.0	Malvidin-3-(6-coumaroylglucoside)	64.25 ± 14.56	4.16 ± 0.34	38.97 ± 2.73	3.24 ± 1.18

Data expressed as mean ± standard deviation. Concentrations expressed as micrograms of the compound per grames of fresh weight (FW); * below the limit of quantification.

Table S2. Concentrations of all compounds found in seedless grape Autumn crisp.at each stage of digestion.

Rt	*Compound*	*Raw material (µg/g FW)*	*Digestion (µg/g FW)*		
			Oral phase	*Gastric phase*	*Intestinal phase*
5.8	Gallic acid	0.38 ± 0.06	1.09 ± 0.94	1.44 ± 0.59	5.65 ± 0.75
9.5	Triptophan	94.50 ± 15.26	87.6 ± 29.21	89.15 ± 39.08	17996.7 ± 3061.68
12.5	Procyanidin B1	1.37 ± 0.3	0.45 ± 0.64	2.15 ± 0.01	33.07 ± 15
13.7	Catechin	2.24 ± 0.18	1.65 ± 0.02	1.96 ± 0.11	30.23 ± 25.58
16.7	Epicatechin	1.6 ± 0.11	1.54 ± 0.15	2.44 ± 0.59	16.96 ± 10.2
25.3	Quercetin-3-glucoside	1.39 ± 0.17	2.07 ± 1.89	3.85	4.11
25.9	Quercetin-3-glucoronide	2.66 ± 0.46	1.32 ± 0.7	2.98	*

Data expressed as mean ± standard deviation. Concentrations expressed as micrograms of the compound per grames of fresh weight (FW); * below the limit of quantification.

Table S3. Concentrations of all compounds found in seedless grape Pristine at each stage of digestion.

Rt	*Compound*	*Raw material (µg/g FM)*	*Digestion (µg/g FW)*		
			Oral phase	*Gastric phase*	*Intestinal phase*
5.7	Gallic acid	1.06 ± 0.89	0.32 ± 0.1	1.3 ± 2.1	5.45 ± 2.6
8.7	Gallocatechin	2.28 ± 0.48	0.96 ± 1.36	*	3.23 ± 1.57
9.3	Triptophan	16.86 ± 1.31	21.35 ± 0.92	36.08 ± 26.1	13979.26 ± 9844.1
12.5	Procyanidin B1	3.66 ± 1.01	0.4 ± 0.57	1.18 ± 1.66	17.62 ± 7.31
13.5	Catechin	4.63 ± 0.6	2.14 ± 0.08	2.08 ± 1.81	11.86 ± 6.31
15.4	Epigalocatechin gallate	2.56 ± 1.58	1.19 ± 0.22	2.21 ± 0.9	3.31 ± 2.1
16.7	Epicatechin	3.19 ± 0.45	2.91 ± 0.34	3.29 ± 1.35	6.68 ± 3.59
25.3	Quercetin-3-glucoside	1.74 ± 0.08	4.29 ± 4.49	6.87	2.13
25.9	Quercetin-3-glucoronide	2.36 ± 0.79	1.98 ± 1.03	2.03	3.01

Data expressed as mean ± standard deviation. Concentrations expressed as micrograms of the compound per grames of fresh weight (FW); * below the limit of quantification.

Table S4. Concentrations of all compounds found in seedless grape Scarlotta at each stage of digestion.

Rt	*Compound*	*Raw material (µg/g FW)*	*Digestion (µg/g FW)*		
			Oral phase	*Gastric phase*	*Intestinal phase*
5.8	Gallic acid	0.76 ± 0.24	0.37	0.69 ± 0.09	4.49 ± 2.65
9.3	Delphinidin-3-glucoside	0.68 ± 0.17	0.43	*	*
9.7	Triptophan	47.86 ± 9.4	54.63 ± 38.72	86.21 ± 4.36	17454.84 ± 4284.19
11.4	Cyanidina-3-glucoside	1.46 ± 2.67	1.13	2.61 ± 0.85	0.5
13.5	Catechin	12.93 ± 4.92	2.28 ± 0.72	2.62 ± 0.26	32.68 ± 2.41
13.9	Peonidin-3-glucoside	21.25 ± 10.39	25.49 ± 12.45	52.31 ± 7.72	7.39 ± 3
14.5	Malvidin-3-glucoside	12.47 ± 6.09	*	*	*
24.7	Malvidin-3-caffeoylglucoside	0.49 ± 0.28	0.49 ± 0.23	1.26 ± 0.89	0.37
25.0	quercetina-3-glucoside	13.68 ± 1.39	1.7 ± 0.61	2.58 ± 0.78	3.38 ± 0.12
25.7	quercetina-3-glucoronide	35.68 ± 1.22	1.57 ± 0.67	2.55 ± 0.82	2.80 ± 1.22
26.0	Peonidin-3-(6- coumaroylglucoside)	1.46 ± 0.68	1.13 ± 0.66	2.61 ± 2.8	0.5 ± 0.22
27.5	Malvidin-3-(6- coumaroylglucoside)	3.92 ± 2.23	1.65 ± 1.28	4.52 ± 4.05	0.49 ± 0.23

Data expressed as mean ± standard deviation. Concentrations expressed as micrograms of the compound per grames of fresh weight (FW); * below the limit of quantification.

Table S5. Concentrations of all compounds found in seedless grape Crimson at each stage of digestion.

Rt	*Compound*	*Raw material (µg/g FW)*	*Digestion (µg/g FW)*		
			Oral phase	*Gastric phase*	*Intestinal phase*
5.7	Gallic acid	1.17 ± 0.38	1.72 ± 1.16	1.23	4.73 ± 0.15
9.7	Triptophan	338.76 ± 28.03	563.25 ± 186.93	413.41 ± 21.57	18474.78 ± 3090.21
11.9	Cyanidina-3-glucoside	3.07 ± 0.56	*	*	*
13.8	Catechin	4.17 ± 0.46	2.01 ± 0.27	3.14 ± 0.53	28.73 ± 2.56
14.0	Peonidin-3-glucoside	33.88 ± 15.59	35.16 ± 27.37	34.23 ± 9.28	32.79 ± 37.83
14.7	Malvidin-3-glucoside	11.86 ± 5.62	*	*	*
15.9	Epigallocatechin gallate	2.7 ± 0.38	1.42 ± 0.67	*	5.17 ± 0.44
16.9	Epicatechin	3.93 ± 0.12	2.4 ± 0.68	2.57 ± 0.17	13.59 ± 9.45
25.6	Quercetina-3-glucoside	3.4 ± 0.49	3.25 ± 1.36	3.44 ± 0.74	3.43 ± 1.71
26.0	Quercitina-3-glucoronoide	1.09 ± 0.14	2.05	1.59 ± 0.61	1.94
27.0	Peonidin-3-(6- coumaroylglucoside)	1.08 ± 0.41	0.74	0.66	*
28.4	Malvidin-3-(6- coumaroylglucoside)	5.47 ± 2.47	0.69 ± 0.42	1.38 ± 0.03	0.91

Data expressed as mean ± standard desviation. Concentration expressed as micrograms of the compound per grames of fresh weight (FW); * belloe the limit of quantification.

Table S6. Concentrations of all compounds found in seedless grape Melody at each stage of digestion.

Rt	*Compound*	*Raw material (µg/g FW)*	*Digestion (µg/g FW)*		
			Oral phase	*Gastric phase*	*Intestinal phase*
5.7	Gallic acid	0.72 ± 0.1	0.76 ± 0.07	0.92	4.35 ± 1.33
9.5	Delphinidin-3-glucoside	11.37 ± 4.73	3.79	6.39 ± 4.08	0.74
9.7	Triptophan	91.02 ± 36.15	76.87 ± 52.23	105.29 ± 35.48	20927.85 ± 3534.92
12.1	Cyanidin-3-glucoside	15.85 ± 6.14	3.5 ± 4.36	11.19 ± 9.63	0.88
12.5	Procyanidin B1	5.01 ± 1.64	*	11.76	*
13.5	Catechin	10.75 ± 4.01	5.27 ± 2.49	9.69 ± 2.97	11.75 ± 3.83
14.0	Peonidin-3-glucoside	12.18 ± 3.79	*	*	*
14.5	Malvidin-3-glucoside	231.44 ± 55.78	51.75 ± 27.9	135.82 ± 55.78	26.73 ± 6.82
16.7	Epicatechin	7.16 ± 1.55	5.18 ± 1.2	6.44 ± 0.74	15.85 ± 2.89
20.5	Myricetin-3-glucoside	30.23 ± 9.12	5.24 ± 1.86	12.84 ± 7	6.5 ± 0.16
21.9	Malvidin-3-acetylglucoside	8.92 ± 1.63	1.83	5.02 ± 0.62	0.55 ± 0.45
24.3	Malvidin-3-caffeoylglucoside	1.41 ± 0.27	1.31 ± 1.3	2.02 ± 0.23	0.51 ± 0.47
25.3	Quercetin-3-glucoside	4.75 ± 1.95	2.75 ± 0.29	3.48 ± 0.34	5.93 ± 1.17
25.6	Quercetin-3-glucoronide	4.69 ± 2.54	1.96 ± 0.49	3.24	5.44
26.6	Peonidin-3-(6-coumaroylglucoside)	23.81 ± 3.11	1.56 ± 0.94	6.77 ± 0.34	0.5 ± 0.46
27.8	Malvidin-3-coumaroylglucoside	58.36 ± 14.99	2.32 ± 0.68	6.25 ± 6.92	0.58
29.0	Kaempherol-3-glucoside	1.56 ± 0.29	*	4.85	*

Data expressed as mean ± standard desviation. Concentration expressed as micrograms of the compound per grames of fresh weight (FW); * belloe the limit of quantification.
